# Essential Oil Composition Analysis, Antimicrobial Activities, and Biosystematic Studies on Six Species of *Salvia*

**DOI:** 10.3390/life13030634

**Published:** 2023-02-24

**Authors:** Azize Demirpolat

**Affiliations:** Vocational School of Food, Agriculture, and Livestock, Bingol University, Bingol 12000, Turkey; ademirpolat@bingol.edu.tr

**Keywords:** chemical constituents, antimicrobial, pollen, *Salvia*, LM-SEM

## Abstract

The essential oil constituents, antimicrobial properties, and biosystematic characteristics (morphological, palynological, and anatomical features) of six *Salvia* species from different regions of Turkey were investigated qualitatively and quantitatively in this study. The chemical composition of the essential oils of dried aerial parts of *Salvia* species, i.e., *S. absconditiflora*, *S. ceratophylla*, *S. multicaulis*, *S. verbenaca*, *S. viridis*, and *S. syriaca* were analyzed by GC-MS. The main constituents of the six *Salvia* species studied were 1,8-cineol, caryophyllene oxide, spathulenol, and borneol in different ratios. The antimicrobial activity of the essential oil extracted from the aerial parts of species of the genus *Salvia* was tested by the disc diffusion method. The essential oils of *Salvia* species showed different antimicrobial activity against the studied microorganisms. The highest antimicrobial activity against *E. coli* was observed in *S. multicaulis* and the highest antimicrobial activity against *K. pneumoniae* was observed in *S. verbenaca*. The morphology of the stem, leaf, bract, and flower structures of the *Salvia* species were analyzed in this study. Anatomical investigations focused on the root, petiole, and stem in more detail. Our research has broadened the criteria of anatomical characters unique to the *Salvia* species. Under light microscopy, the pollen grains of the six species belonging to *Salvia* were isopolar and radially symmetrical. The properties of the essential oil constituents, antimicrobial properties, and biosystematic data obtained in this study contribute to the bioactive and biosystematic studies of *Salvia* species used for food, pharmaceutical, and cosmetic purposes.

## 1. Introduction

*Salvia* L. gets its name from the Latin word “Salvare”, which means “to heal or treat” [1]. *Salvia* belongs to the Salvinae subtribe, Lamiaceae family, Lamiales order of dicotyledonous. *Salvia* species have been identified in the group of commercial, medicinal, and aromatic herbs and are one of the various centers in Turkey, especially the Anatolia region [2]. Therefore, Turkey exhibits the greatest expansion of sage and is also one of the countries that widely consume it for commercial purposes [3].

*Salvia* is an important genus in the Lamiaceae family with approximately 1000 species worldwide, which contains many species producing secondary metabolites [4]. According to the most recent Flora of Turkey, there are more than 86 *Salvia* taxa in Turkey, while other studies indicate that it has increased to about 100 species [5]. Turkish *Salvia* species are discussed in seven sections, including *Aethiopis*, *Drymosphace*, *Hymenosphace*, *Horminum*, *Hemisphace*, *Plethiosphace*, and *Salvia*. In the current study, the divisions of the sections are as follows: *S. absconditiflora* (Montbret & Aucher ex Benth.) (syn. *Salvia cryptantha* Montbret & Aucher ex Benth)*, S. multicaulis* Vahl. in the *Hymenosphace* sectionss, *S. syriaca* L., *S. verbenaca* L., and *S. ceratophylla* L. in the *Aethiopis* section, and *S. viridis* L. in the *Horminum* section [2,6,7,8].

Molecular characterization studies carried out in recent years give important ideas about the systematics of species [9,10,11,12,13]. Genetic characterization studies among *Salvia* species have also revealed a large genetic variation among species. Similarly, flow cytometry and genome size determination studies have shown that genetic diversity is very rich among samples collected from different locations of the same species [4].

For thousands of years, the *Salvia* genus has been used in traditional medicine and is utilized in a broad variety of commercial and pharmaceutical goods, particularly in the production of essential or volatile oils and flavoring compounds, as well as in the food and cosmetics sectors. Essential oil components of *Salvia* species have an important place in the medicinal, aromatic plants market. Several studies have been conducted on the essential oil of this genus [14,15,16]. The chemical composition of *Salvia* L. was investigated by Gas Chromatography–Mass Spectrometry system with major compounds including caryophyllene oxide, spathulenol, α-copaene, germacrene D, and *β*-pinene [17]. Despite the fact that the majority of *Salvia* species are employed in traditional herbal medicine, studies have shown that they also exhibit anti-inflammatory, antibacterial, antioxidant, anticancer, and antidiabetic properties [12,14,15,18,19,20,21]. Additionally, these essential oils have been utilized to treat eczema, psoriasis, and asthma diseases [22]. *S. absconditiflora* species, represented in group C in the Flora of Turkey, is an endemic species. These endemic species have been researched and consumed as herbal tea in Turkey [23]. *S. absconditiflora* is thought to have a strong antioxidant effect because of its high phenolic and flavonoid content. Additionally, *S. absconditiflora* was said to have a strong cytotoxic effect on cancer cells [15]. Furthermore, *S. syriaca* is utilized in the food industry, the pharmacology and cosmetic industry, and it is also used to treat animals [24]. Moreover, *S. ceratophylla* is traditionally consumed to cure microbial infections, cancer, and urinary tract problems in Jordan [25,26]. However, some *Salvia* species have antimicrobial activity when extracted from above-ground soil [27,28].

Some *Salvia* species were compared with anatomical, morphological, and pollen features [29] and the findings showed that there were similarities and differences among the species. Thus, several of these important features, including the form of the calyx, corolla, and stamen, may be utilized to discriminate across infrageneric categories. Calyx and corolla shape, bract morphology and structure as well as inflorescence type are important diagnostic characteristics of taxonomic value regarding the six studied *Salvia* species, i.e., *S. absconditiflora*, *S. ceratophylla*, *S. multicaulis*, *S. verbenaca*, *S. viridis*, and *S. syriaca*. The morphology of the stem, leaf, bract, and flower structures of the *Salvia* species were analyzed in this study. Pollen characteristics of the family Lamiaceae have considerable taxonomic importance and the classification of genera in Labiatae has been revised [30,31], with *Salvia* placed within the subfamily Nepetoideae because it had hexacolpate pollen [29].

This study aimed to contribute to both the systematics and bioactivity of the genus by investigating the qualitative and quantitative characteristics of some biosystematic features (morphological, palynological, and anatomical features) as well as the essential oil constituents and antimicrobial properties of six *Salvia* plants collected from different regions of Turkey.

## 2. Materials and Methods

### 2.1. Plant Material

Plant samples were collected from the locations indicated in Table 1. All plants were collected during the flowering period and in the morning hours [32]. For essential oil studies, approximately 200 g of plant samples were dried in a place out of the sun. For anatomical studies, the plants were stored in 70% alcohol in the area where they were collected. For morphological and palynological examinations, 20 plant samples were selected from each taxon; the samples were dried and kept as herbarium specimens at Bingol University.

### 2.2. Isolation of Essential Oils and GC-MS Analysis

The plants used in this research were air-dried. The oil from the plants was extracted using the hydrodistillation technique. Using Clevenger equipment, three hours of hydrodistillation were performed on the 100 g of air-dried aerial plant materials. The organic layer in the gathering vial was transferred into the GC/GC-MS equipment once the distillation process was accomplished.

The GC-MS was used to examine the essential oil. The instrument was an HP 6890 model. The mass range was between 40 and 330 m/z, and the ionization energy was 70 eV. A column HP-5 MS (30 m 0.25 mm i.d., film thickness 0.25 m) capillary column with a column flow rate (transporter gas helium) was implemented. Helium is used as the carrier gas, with a steady column flow rate of 1 mL/min. The settings for the Column Oven Temperature procedure were 40 °C and a hold time of 2 min at a temperature of 3 °C/min. 240 °C was the final degree. The flow rate was set to 1 L, and the split mode was chosen (split ratio 1:10 or 1:100). A 3.5 min buffer hold was applied to hexane samples.

The mass spectrometric settings were full scan mode, 20,000 amu/s scan speed, and 50 spectra per sample frequency. Temperatures at the contact and ion source were 250 °C and 200 °C, etc.

Alkanes were used as standards to compute the retention indices (RI). By comparing the retention times (RT), mass spectra, and RI of the essential oils to those described in the literature (NIST 20 and Wiley Libraries) and MS libraries (Wiley), the chemical components of the essential oils were identified. Traditional library searches just compare spectra rather than taking retention parameters into account. In this study, libraries were searched using a combination of storage indexes, which made compound identification simpler and more accurate. The device’s retention index spectrum libraries were also utilized in this study. The same analytical procedure as the identical column provided in the library was applied for better results. Table 2 details the essential oil constituents that have been identified.

### 2.3. PCA (Principal Component Analysis)

**Figure 1:**
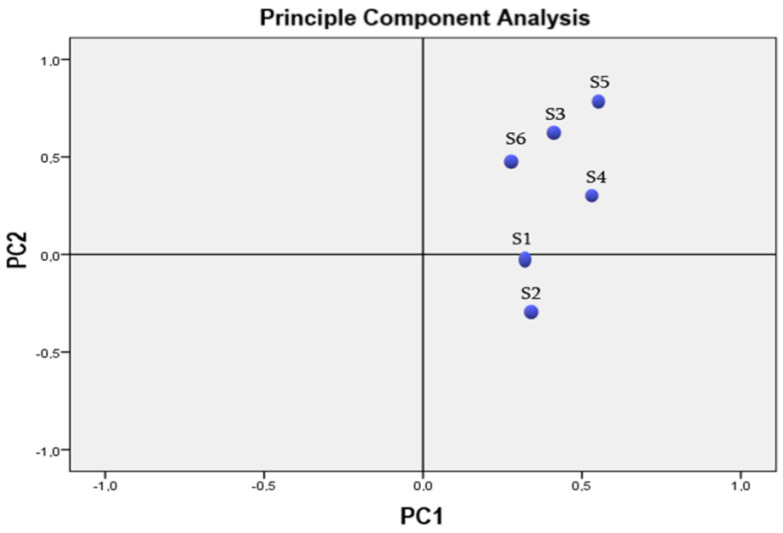
The principal component analysis (PCA) of the essential-oil composition of *Salvia* species.

Multivariate analysis was performed to determine the structure of variability and to calculate differences between groups. Complete data sets were used for these analyses. To determine the commonalities between the measurement units, the UPGMA (unweighted pair-group average linkage) clustering approach based on Pearson distances was used (Figure 1). The chemical components of the essential oils of the *Salvia* species were considered as variables. The chemical components of the different samples were evaluated using cluster analysis (CA) and principal component analysis (PCA). The same weight was given to the non-standardized statistics as previously reported.

### 2.4. Antimicrobial Investigations

**Figure 2:**
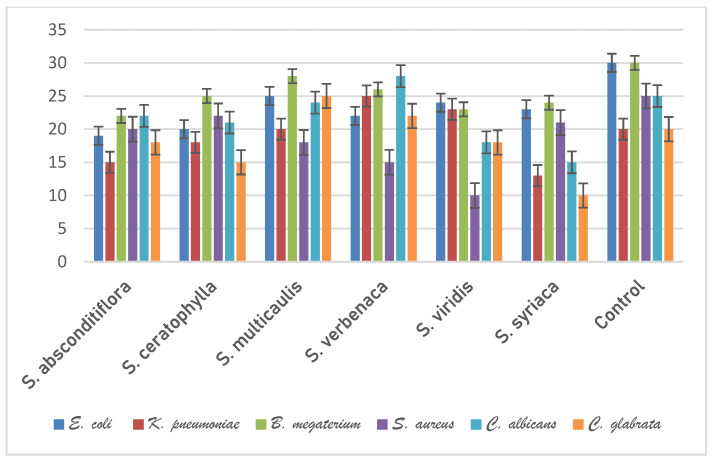
Graph showing the antimicrobial effects of six *Salvia* essential oils against test microorganisms.

The disk diffusion technique was used to test the plant essential oil’s antibacterial activity [36]. Yeast strains (*Candida albicans* and *Candida glabrata*) were cultured in malt extract broth for 48 h at 25 ± 1 °C, whereas bacteria strains (*Escherichia coli*, *Klebsiella pneumoniae*, *Staphylococcus aureus*, and *Bacillus megaterium*) were incubated in nutrient broth for 24 h at 35 ± 1 °C. At a rate of 1%, the bacteria and yeast cultures made in broth were added to Mueller Hinton Agar and Plate Count Agar, respectively (10^6^ bacteria mL, 10^4^ yeast mL, 10^4^ fungi mL^−1^). 25 cc of the cultures were added to sterile 9 cm diameter petri plates after being well shaken. The medium’s homogeneous dispersion was accomplished. On the solidified agar medium, antimicrobial discs with a 6 mm diameter that were each impregnated with 100 μL of essential oil were lightly positioned. The plates infected with bacteria were incubated at 37 ± 0.1 °C for 24 h and the plates inoculated with yeast were incubated at 25 ± 0.1 °C for 72 h after being kept the petri dishes generated in this manner at 4 °C for 1.5 to 2 h. Different standard discs were used as controls for yeasts (Nystatin 100 mg/disc) and bacteria (Streptomycin sulfate 10 mg/disc). Inhibitory zones were measured in millimeters (Figure 2).

### 2.5. Morphological Investigations

The taxonomic characters of the *Salvia* species were prepared in 20 samples according to Flora of Turkey and some significant articles [2,5,6]. The morphological and morphometrical characters are presented in the result section. Morphological measurements were taken with calipers. All of the morphological measurements were performed using Hierarchical clustering analysis using SPSS software version 21, and the resulting dendrogram was shown.

### 2.6. Anatomical Investigations

Anatomical investigations were conducted using an average of twenty specimens unbroken in 70% alcohol. The cross-sections of stem, root, and petiole organs were cut with a razor, and sections were stained with Alcian blue for cellulose substances and safranine O for polymer substances within the quantitative relation of 3:2. For staining, the sections were placed in the ready dye for five minutes [37,38]. Furthermore, sections were examined and measured using a Euromex CMEX-10 PRO light microscope.

### 2.7. Palynological Investigations

**Figure 3:**
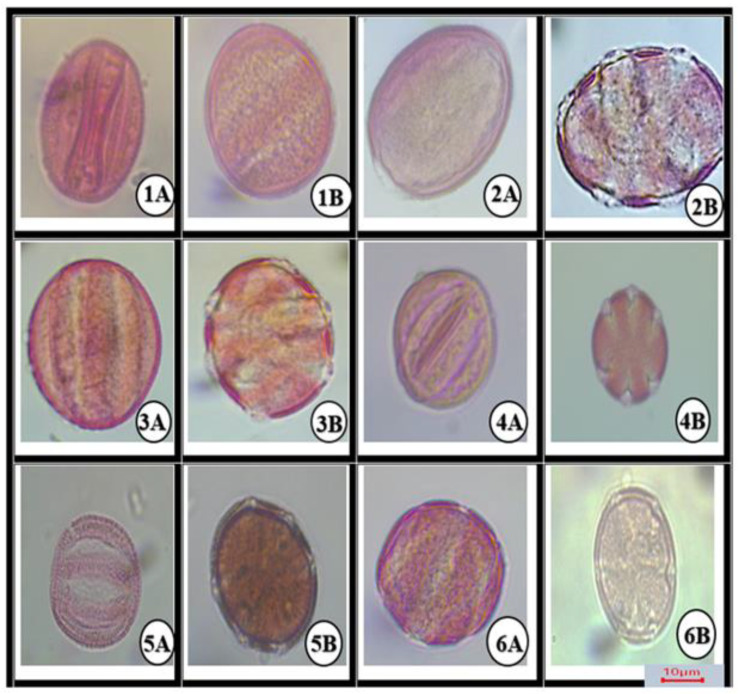
Light microscope microphotographs at 1000× magnification. A: equatorial view, B: polar view, 1: *S. absconditiflora*, 2: *S. ceratophylla*, 3: *S. multicaulis*, 4: *S. verbenaca*, 5: *S. viridis*, 6: *S. syriaca*.

Pollens were generated using the Ertman technique, using samples from the light microscopy [30]. A few changes were made to the acetolysis procedure after then. Plant matter was cursed for one to two minutes using a glass rod and one to two drops of acetic acid in the slide. A needle was used to clear the particles off the slide. A coverslip was placed over the slide after applying a drop of glycerin jelly. Each specimen received 4–5 prepared slides in total. Polar axis (P), equatorial axis (E), colpus length (Clg), colpus width (Clt), exine thickness (Ex), and aperture width (Ap) were measured from at least 20 completely evolved grains according to the pattern beneath a Euromex CMEX-10 PRO light microscope (100×). These measurements are reported in Table 3, and micrographs in Figure 3. The terminology used is mainly from Faegri & Iversen, Ertman, and the study of Kılıç et al. [39,40,41].

**Figure 4:**
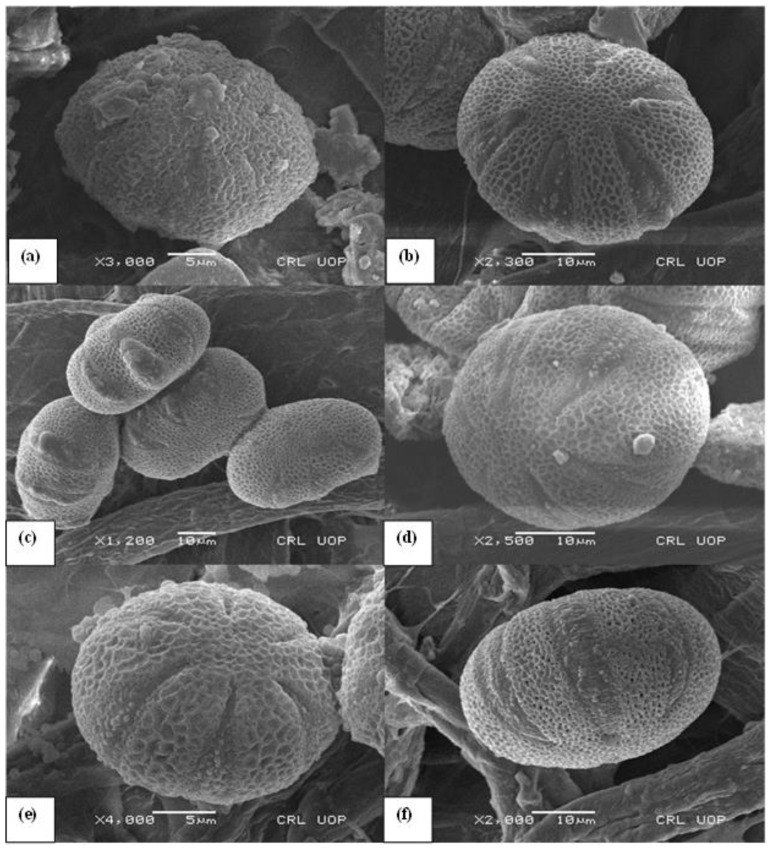
Scanning electron micrographs of exine ultrasculpture view, (**a**) *S. absconditiflora*, (**b**) *S. ceratophylla*, (**c**) *S. multicaulis*, (**d**) *S. verbenaca*, (**e**) *S. viridis*, (**f**) *S. syriaca*.

For scanning electron microscopy (SEM) *Salvia* species pollen slides were prepared using the techniques of Majeed et al. [42]. Pollen was acetolyzed before being suspended in 90% ethanol for SEM. They were then placed on metallic stubs that had been gold-palladium coated. Pollen electron micrographs are taken with an SEM (Model JEOL JSM5910). Results from pollen SEM are summarized in Figure 4.

**Figure 5:**
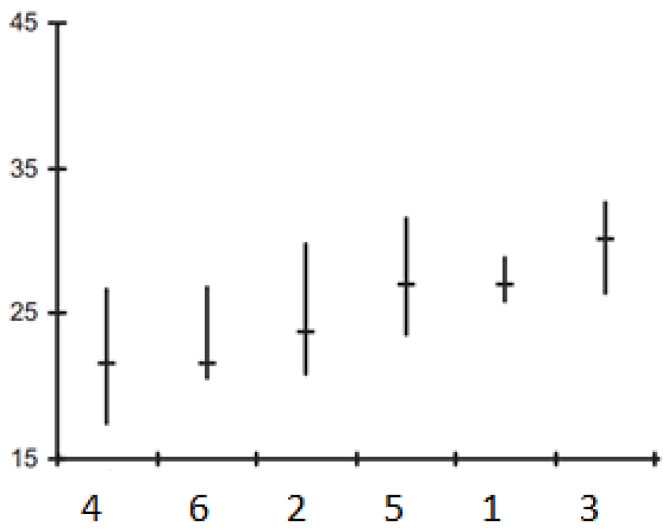
The maximum, minimum, and average size of the polar axis (P) of *Salvia* species (1: *S. absconditiflora*, 2: *S. ceratophylla*, 3: *S. multicaulis*, 4: *S. verbenaca*, 5: *S.viridis*, and 6: *S.syriaca*).

**Figure 6:**
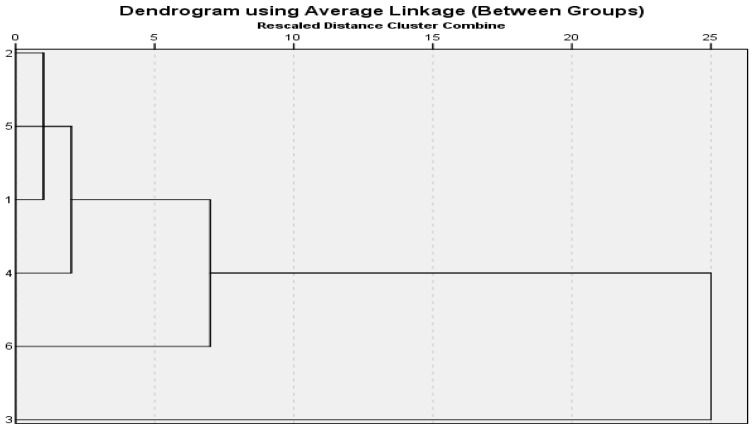
The cluster analysis of palynological data of *Salvia* species (1: *S. absconditiflora*, 2: *S. ceratophylla*, 3: *S. multicaulis*, 4: *S. verbenaca*, 5: *S. viridis*, 6: *S. syriaca*).

All of the pollen measurements were performed using Hierarchical clustering analysis performed using SPSS software version 21 and the resulting dendrogram was shown in Figure 5 and Figure 6.

## 3. Results

### 3.1. Essential Oil Components

Qualitative and quantitative differences were found in the essential oil analysis of the six *Salvia* species the essential oils of *S. absconditiflora*, and *S. ceratophylla* 29, and 28 components were identified representing 100% and 98.62% of the oils, respectively. The aerial part of *S. absconditiflora* and *S. ceratophylla* were hydrodistilled, obtaining yields of 0.97% and 0.75% (*w*/*w*) of yellowish oils, respectively. The aerial parts of the *S. multicaulis* and *S. verbenaca* were hydrodistilled, obtaining yields of 0.97% and 0.95% (*w*/*w*) of yellowish oils, respectively. In the essential oils of this species, 27 and 25 components were identified representing 92.60% and 94.26% of the oils, respectively. *S. viridis* has 30 components (0.78 *w*/*w*). The essential oils of *S.viridis* have 29 components and *S. syriaca* was identified with 31 components. Additionally, this species was representing 91.54% and 91.02% of the oils, respectively.

The major compounds were 1,8-cineol (17.94%), borneol (10.40%), caryophyllene oxide (10.14%), spathulenol (9.09%), and caryophyllene (8.45%) in *S. absconditiflora*; spathulenol (20.13%), caryophyllene oxide (14.68%), 1,8-cineol (12.98%), and caryophyllene (8.36%) in *S. ceratophylla*; spathulenol (18.10%), caryophyllene oxide (17.20%), 1,8-cineol (11.99%), bicyclogermacrene (5.89%), borneol (5.74%), and caryophyllene (8.51%) in *S. multicaulis*; caryophyllene oxide (16.15%), spathulenol (13.18%), 1,8-cineol (11.45%), bicyclogermacrene (11.03%), and borneol (11.00%) in *S. verbeneca*; caryophyllene oxide (16.18%), caryophyllene (15.01%), 1,8-cineol (14.06%), spathulenol (11.42%), β-pinene (7.21%), and borneol (7.02%) in *S. viridis*; caryophyllene oxide (17.54%), spathulenol (9.35%), borneol (9.65%), bicyclogermacrene (6.93%), and 1,5-epoxy*salvia*l-4[14]-ene (6.83%) in *S. syriaca*. The compositions of six of the *Salvia* essential oils are listed in Table 2.

Based on other research that has been published, multivariate analysis was employed [43]. The chemicals for the various samples were identified using principal component analysis (PCA) and cluster analysis (CA). The PCA was then carried out using the matrix correlation setup and Varimax rotation. PC1 (48.13%) and PC2 (10.04%) were the primary components in the principal component analysis. The total load of PC1 and PC2 was 58.17%. The Kaiser-Meyer-Olkin (KMO) approach was used to investigate the correlation of the variables. KMO was 0.613, which is considered satisfactory. Barlett’s test of sphericity indicated statistical significance at alpha 0.06 for the data set. PCA analysis was explained in two ways, which revealed the link between the six *Salvia* species and their essential oil concentration (Figure 1).

### 3.2. Antimicrobial Activity Studies

In this step of the study, the antimicrobial activity of the essential oil obtained from the above-ground parts of six species belonging to the genus *Salvia* was tested by the disc diffusion method. Antimicrobial activity was tested against *E. coli*, *K. pneumoniae*, *B. megaterium*, *S. aureus* bacteria, *C. albicans*, and *C. glabrata* yeasts. Streptomycin sulphate 10 µg/disc for bacteria and Nystatin 100 µg/disc for yeasts were used as controls.

Essential oils of *Salvia* species showed varying antimicrobial activity against the microorganisms studied. The highest antimicrobial effect against *E. coli* was observed in *S. multicaulis* (25 mm), while the lowest antimicrobial effect was observed in *S. absconditiflora* (19 mm). The highest antimicrobial effect against *K. pneumoniae* was observed in *S. verbenaca* (25 mm), while the lowest antimicrobial effect was observed in *S. syriaca* (13 mm). The highest antimicrobial effect against *B. megaterium* was observed in *S. multicaulis* (28 mm), while the lowest antimicrobial effect was observed in *S. absconditiflora* (22 mm). The highest antimicrobial effect against *S. aureus* was observed in *S. ceratophylla* (22 mm), while the lowest antimicrobial effect was observed in *S. viridis* (10 mm). The highest antimicrobial effect against *C. albicans* was observed in *S. verbenaca* (28 mm), while the lowest antimicrobial effect was observed in *S. syriaca* (15 mm). The highest antimicrobial effect against *C. glabrata* was observed in *S. multicaulis* (25 mm), while the lowest antimicrobial effect was observed in *S. syriaca* (10 mm). According to these results, *S. multicaulis* and *S. verbenaca* species had the strongest antimicrobial activity, while *S. absconditiflora* and *S. syriaca* had the lowest activity. The antimicrobial activity of the plant samples against the test microorganisms is shown graphically in Figure 2.

### 3.3. Morphology Properties

**Figure 7:**
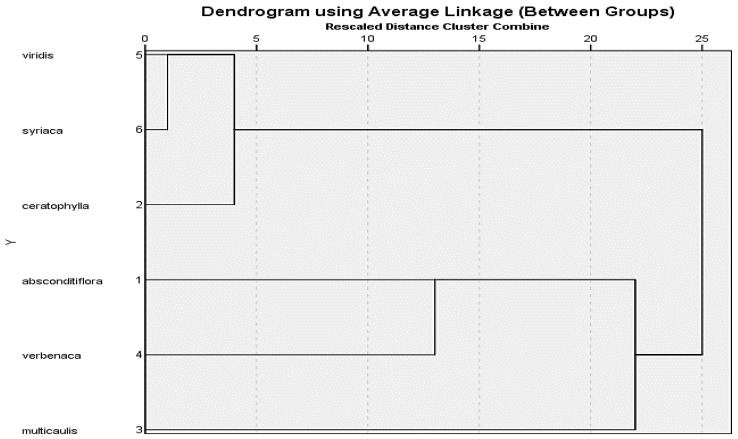
The cluster analysis of *Salvia* species in this study, according to morphology.

**Figure 8:**
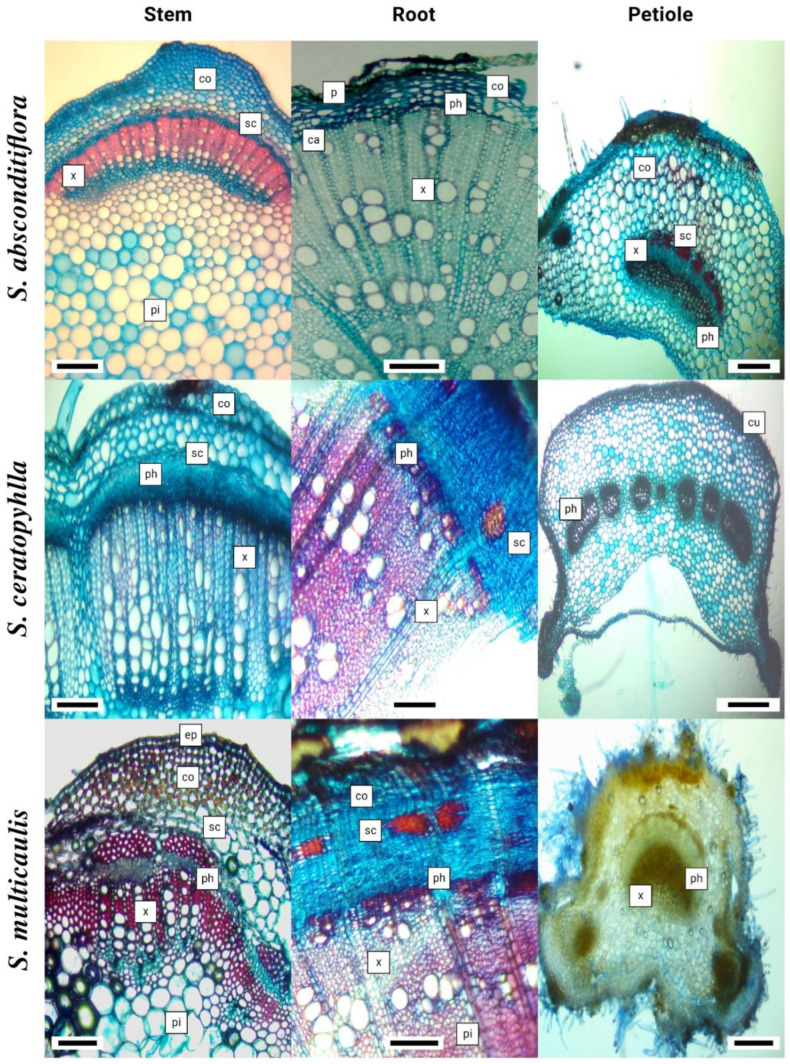
Light Microscopy: Stem, root, and petiole cross-section of the *Salvia* species (ep: epidermis, co-col: collenchyma, co: cortex, ca: cambium, x: xylem, ph: phloem, p: pith region, p: para periderma, cu: cuticle).

Morphological observations and measurements of the studied *Salvia* species were made from herbarium specimens. Stem lengths, leaf measurements and characters, calyx and corolla characteristics and measurements, petiole measurements, inflorescence types, and hair conditions of the studied six *Salvia* species were determined. The endemic species *S. absconditiflora* was a perennial herb with elliptical cordate leaves, whose habitats were roadsides, uncultivated fields, slopes, and rocky limestone. The habitat of *S. ceratophylla* is mud and inactive, limestone rocky areas. The stem of this biennial species is erect and strong and has dense glandular hairs. The two perennial species *S. multicaulis* and *S. verbenaca* are very similar to each other (Figure 7), but differ in that S. *verbenaca* is densely hairy. *S. multicaulis* have hair on the body pilose to villous, rarely glabrous, sometimes dendroid hairy. *S. viridis* was an annual plant and its habitats were rocky slopes. *S. syriaca* was a perennial herb, rhizomatous. The stem was upright branches, and glandular feathers are quite dense. Descriptions of morphological and morphometric characters are described in Table 4, Figure 7 on the six *Salvia* species. All of the morphological measurements were performed using hierarchical clustering analysis and the resulting dendrogram was shown in Figure 7. Two large clusters were formed as a result of clustering analysis. *S. absconditiflora*, *S. multicaulis*, and *S. verbenaca* species are located on one side of the cluster (Figure 7). *S. multicaulis*, which is in the outermost clade, and *S. absconditiflora*, which is the closest to it, are species located in the same section in the Flora of Turkey [6] and can be distinguished morphologically by their leaf sizes and the color status of the calyx. The cluster tree in this study confirms these results in terms of morphology in Figure 8.

### 3.4. Anatomical Properties

The stem epidermis of *S. absconditiflora* has a layer of collenchyma embedded in the cortex, usually below a single-layered epidermis. In section through the stem, the pith covered a large area. In the stem cross-section, most of the cells in the periderm were crushed. Xylem rails were obvious. In the cross-section of the petiole of *S. absconditiflora*, there were two areas named abaxial surface and adaxial surface. The adaxial surface has a convex shape. A cuticle surrounded the petiole. A single row of rectangular and oval cells made up the epidermis. The epidermis was covered with trichomes. The stem of *S. ceratophylla* have located single-layered and made up of cells with an oval-oblong shape. Sclerenchymatic cells consisted of 3–7 layers and were usually stained red color on sections. The root of *S. ceratophylla a* periderm located at the outermost part was dark-colored. Most of the periderm was crushed and its cell structure was disrupted. In the petiole’s cross-section of this species, the adaxial surface was convex. The stem epidermis of *S. multicaulis* has a single-layered epidermis and is made up of cells that were typically oval-oblong and sometimes square-like in shape.

**Figure 9:**
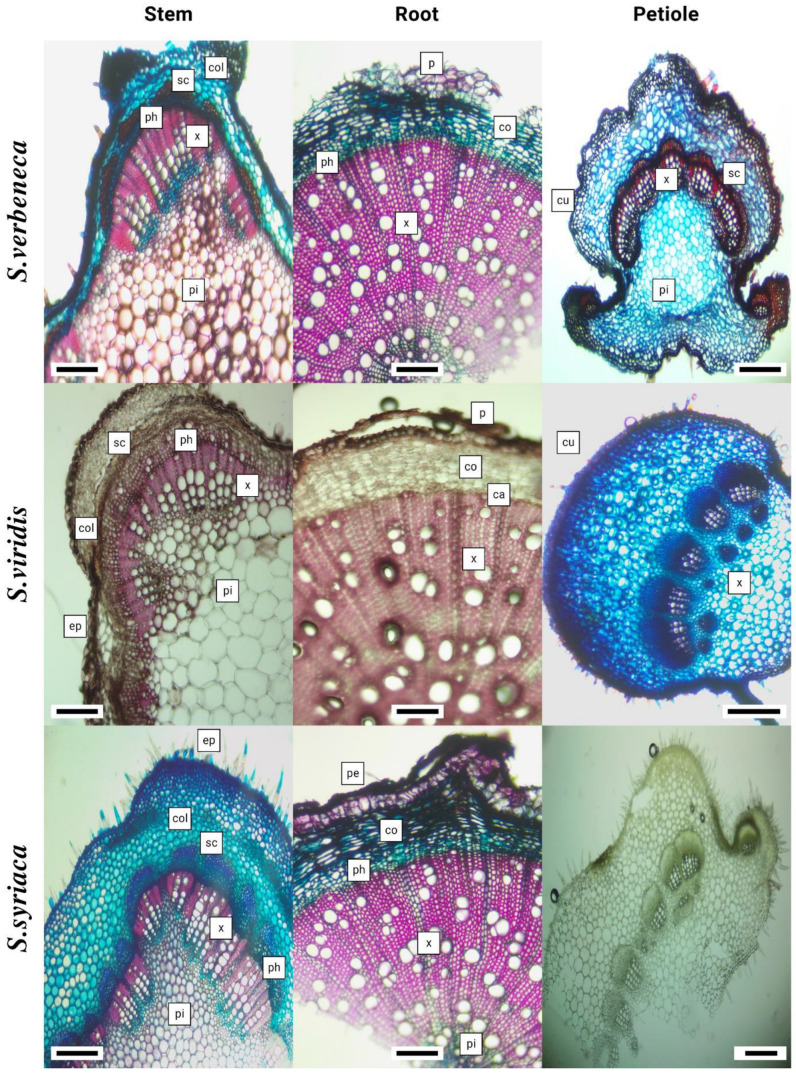
Light Microscopy: Stem, root, and petiole cross-sections of the *Salvia* species (ep: epidermis, co-col: collenchyma, co: cortex, ca: cambium, x: xylem, ph: phloem, p: pith region, p: para periderm).

The stem epidermis of *S. verbenaca* consisted of oval-oblong, sometimes square-like cells and was single-layered. There was a layer of collenchyma in the cortex under the epidermis in certain spots, and both adaxial and abaxial surfaces were convex and twisted in a petiole cross-section. The stem epidermis of *S. viridis* and *S. syriaca* were single-layered and consisted of mostly ovoidal rectangular. When we took a cross-section of the petiole *S. viridis*, the epidermis consisted of a single row of rectangular and oval cells. The epidermis was covered with trichomes. The petiole’s cross-section of *S. syriaca* has an abaxial surface and the adaxial surface were procumbent and D-shaped. Petiole was covered with a cuticle. The epidermis consisted of a single row of rectangular and oval cells. The epidermis was covered with trichomes. Descriptions of anatomical characters are expanded with the detailed investigations on six *Salvia* species in Table 5 and Figure 8 and Figure 9.

### 3.5. Palynological Properties

All morphological parameters determined have been shown in Table 3 and Figure 3, Figure 4, Figure 5 and Figure 6. Under LM, the pollen grains of the 6 belonging to *Salvia* were isopolar and radially symmetrical. The pollen was symmetrical relative to the equatorial diameter.

Pollen grains of all *Salvia* species found in this study were hexacolpat and also reticulated ornamentation was observed. The polar axis (P) ranged from 34.2 ± (0.6) μm to 57.2 ± (2.7) μm and the equatorial axis (E) ranged from 29.2 ± (1.2) to 55.3 ± (1.2) μm. The polar axis was longest in *S. multicaulis* 57.2 ± (2.7) μm and shortest in *S. verbenaca* 34.2 ± (0.6) μm (Figure 4). The equatorial axis was longest in *S. multicaulis* 55.3 ± (1.2) μm and shortest in *S. verbenaca* 29.2 ± (1.2) μm.

Clt ratios of all *Salvia* species examined were similar. Exine thickness ranged from 1.2 to 1.9 ± (0.6/0.2) μm. Colpus length varied from 23.2 ± (0.9) μm in *S. verbenaca* to 38.6 ± (3.10) μm in *S. multicaulis*. Colpus width varied from 2.5 ± (1–2) μm in *S. verbenaca* to 6.4 ± (0.9) μm in *S. multicaulis* (Table 3). The length of the colpus and the length of the polar axis are linked in a controlled manner (Figure 3).

*S. absconditiflora*, *S. ceratophylla*, and *S. viridis* species are closest to each other in cluster analysis in Figure 4. *S. multicaulis* species is the most in the outermost clade. A P/E ratio of 1.03 prolate-spheroidal has the largest pollen in this study. This cluster analysis has been carried out correctly to provide this data.

## 4. Discussion

The essential oil constituents and antimicrobial properties as well as some biosystematic characteristics (morphological, palynological, and anatomical features) of *Salvia* samples from different regions of Turkey were studied qualitatively and quantitatively.

In morphological examinations, calyx and corolla shape, bract structure, and inflorescence status are important characters in determining the species included in the six *Salvia* species. No new characters other than those described in the literature concerning the morphological traits of the species that served as the focus of this study were discovered. It was observed that the morphological measurement values of the samples belonging to the *Salvia* species were in great agreement with the findings of the literature [6], as well as some deviations in the minimum and maximum limits of the measurement values. For example, when morphologically examined, the bracts and leaves of the *S. viridis* species were measured smaller than the Flora of Turkey [6] in this study. *S. multicaulis* stem length was measured as smaller and leaves were larger in this study when compared to the Flora of Turkey. The species we collected were taken from a higher altitude compared to the Flora of Turkey. These differences are also observed in Figure 4 and the morphological distinction of the species from each other is indicated in the cluster analysis. When the morphological measurements were compared with the literature, the reason for these differences can be attributed to the difference in the number of samples examined and the place and time of collection. Baran reported that leaf size was 1.2–6 × 0.6–2.8 cm and the corolla size was 0.9–1.5 cm in *Salvia viridis* [44]. The findings obtained in this study showed that the leaves were 1.5–2.5 × 1–3 cm in size and simple, oblong-ovate; corolla size 8–10 mm.

The result of this study is important regarding the usability of 1,8-cineol, caryophyllene oxide, spathulenol, and borneol, which are the major components of *Salvia* species. In a study, *γ*-muurolene (11.4%) and α-pinene (7.6%) were determined as the main compounds in *S. ceratophylla* essential oil [45]. According to the results of this study, no γ-muurolene compound was found in *S. ceratophylla*, while the *α*-pinene ratio was determined as (3.76%). As a result of essential oil component analyses of *S. multicaulis* samples carried out by different researchers, different components were reported [46,47,48]. The essential oil obtained from the flowering shoots of *S. multicaulis* was found to be very valuable. The main components of this essential oil were reported to be bornyl acetate, β-caryophyllene, *α*-pinene, camphor, *α*-copaene, myrtenol, sabinyl acetate, 1,8-cineole, limonene, borneol [48,49,50]. In this study, the major components found in the essential oil obtained from *S. multicaulis* were spathulenol (18.10%), caryophyllene oxide (17.20%), 1,8-cineol (11.99%), bicyclogermacrene (5.89%), borneol (5.74%), and caryophyllene (8.51%).

In a previous study, the major constituents of the essential oil of *S. cryptantha S. absconditiflora*, collected from different locations, were 1,8-cineole (21%), camphor (19.1%), α-pinene (12.5%), and camphene (8.7%), while *S. syriaca* contained spathulenol (24.96%), borneol (12.73%), camphene (9.95%), and caryophyllene oxide (8.7%) [23,51]. It is thought that ecological, climatic, plant collection periods and methodological differences are effective in different results in different areas. In this study, although the basic components were similar, their amounts varied.

In this study, 1,8-cineole, which is highly present in *Salvia* essential oils, is used as a component of many medicines such as antiseptics, nasal sprays, mouthwashes, cough syrups, medicated lozenges, and as an additive in personal care products such as toothpaste and aromatherapy oils. Due to the pleasant flavor and aroma of the compound, it is used as a sweetener in products such as confectionery, pastry, bakery products, beverages, and meat products [52]. In a study, the efficacy of 1,8-cineole on the antimicrobial effect against some microorganisms was investigated. As a result of the study, it was concluded that the use of 1,8-cineole in combination with chlorhexidine may facilitate the elimination of some resistant bacteria by increasing antimicrobial activity [53]. The primary sesquiterpene in hops, caryophyllene, or its derivatives, are used in soaps and scents for cosmetic purposes [54]. Hops’ modest sedative effects in herbal medicine are caused by the compound caryophyllene. Furthermore, investigations conducted in vitro showed that caryophyllene has lethal effects on breast cancer cells [55]. The caryophyllene oxide levels in this research showed that as follows; *S. absconditiflora* 10.14%, *S. ceratophylla* 14.68%, *S. multicaulis* 17.20%, *S. verbenaca* 16.15%, *S. viridis* 16.18%, and *S. syriaca* 17.54% (Table 2). The detailed oil composition characterization carried out in this study revealed the presence of various valuable compounds in the chosen *Salvia* species demonstrating their applicability for medicinal and pharmaceutical purposes as well as in the cosmetic beverages industry.

Spathulenol, which was determined as the major compound in the study, is a sesquiterpene component found in essential oils. It has been reported to play a major role in antimicrobial, antiproliferative, anti-inflammatory, and immunomodulatory activities [56,57]. It was also found to have a repellent effect against mosquito species [58]. According to the results of this study, all studied species showed high amounts of spathulenol. Borneol, the other major component, is a colthisless, crystalline monoterpene occurring in essential oils. Borneol has been proven to have antibacterial, antifungal, antispasmodic, choleretic, and sedative effects [59,60]. Recent studies have shown that the blood-brain barrier improves drug delivery and increases efficacy [61]. At the same time, it was determined that borneol showed antiapoptotic, antioxidative, and neuroprotective effects in human neuroblastoma cells [62].

The biochemical contents of *Salvia* species, the solvents used and the differences of microorganisms affect the antimicrobial results. This study reveals that the antimicrobial effect of *Salvia* essential oils is very important. In a previous study, the ethanol extract of the species *S. absconditiflora* (*S. cryptantha*) was tested by the disk diffusion method. As a result of the study, the antimicrobial effect of the plant extracts against “gram+” bacteria was found, while the same effect against “gram-” bacteria and *C. albicans* yeast was not found [63]. In this study, the essential oil of *S. absconditiflora* was effective against both “gram+” and “gram-” bacteria. In an antimicrobial study of *S. ceratophylla* extract, it was observed that it showed a strong antimicrobial effect [64]. Previous studies reported that the essential oils of *S. multicaulis* were effective against *S. aureus*, *K. pneumoniae*, *E. coli*, and *Streptococcus* mutants [65]. In another study, the essential oils of *S. multicaulis* were found to be effective against *Bacillus* sp., *Enterococcus* sp., *Staphylococcus* sp., and *Saccharomyces cerevisiae* [66,67]. In another study, disc diffusion of the essential oil of *S. verbenaca* species showed antimicrobial activity against *Bacillus* sp. and *Staphylococcus* sp. [68]. The essential oils obtained in this study were included in antimicrobial activity studies by the disk diffusion method. This is the first study on the antimicrobial activity of *S. absconditiflora*, *S. ceratophylla*, and *S. viridis* species using this method. In the present study, it was determined that the six *Salvia* species could be considered as a natural antimicrobial source against the tested microorganisms.

In an anatomical study on *S. forskaohlei* L., it was determined that there was a sclerenchymatous ring with sclerenchyma clusters under the parenchymatic cortex cells in the root of *S. forskaohlei* [69]. Çobanoğlu mentioned these sclerenchyma clusters in the root cortex of the species in his study on *S. palestina* Bentham [70]. These findings showed that, in anatomical examinations, sclerenchyma clusters in the root cortex of the species were found in *S. ceratophylla* and *S. multicaulis* species and not in other species.

Metcalfe and Chalk [71] stated that the typical feature of the family is the presence of a well-developed collenchyma tissue at the corners of the stem. Thickening of the collenchyma tissue was observed and photographed in the examined *Salvia* species. Kahraman reported that *S. absconditiflora*, *S. viridis*, *S. ceratophylla*, *S. syriaca*, and *S. viridis*, had a very large cortex and the epidermis consisting of a single subcaste of nearly rectangular, square, or round cells [72]. In this study, a large cortex was observed in the stem sections of the species. In addition, the shape of the epidermis was observed in the cross-sections of the stem in this study, usually ovoidal rectangular and sometimes square. In his study, Kahraman was able to categorize the petiole anatomy of *Salvia* species in a cross-section into seven types. He reported that U-shaped with obtuse or erect margins (*S. viridis*), D-shaped with more or less procumbent margins (*S. syriaca*), triangular (*S. absconditiflora*, *S. multicaulis*) or open crescent-shaped.

Özler et al. pronounced that the *Salvia* section’s pollen suboblate to subprolate and aperture circumstance is hexacolpate and octacolpate [73]. In another study, Özler et al. pronounced that the *S. multicaulis* pollen grain is prolate spheroidal. In this study of the Hymenosphace section, *S. absconditiflora* pollen grain is prolate spheroidal, and *S. multicaulis* pollen grain is suboblate [73,74].

The findings obtained in this study showed that *S. syriaca*, *S. verbenaca*, and *S. ceratophylla* in the *Aethiopis* section species pollen are subprolate, subprolate, and suboblate, respectively. Kiliç reported that *S. syriaca* pollen is suboblate [75]. Moon et al. [76] reported bireticulate ornamentation in pollen of the Aethiopis section, and another study discovered that *S. syriaca* was characterized by reticulate-perforate [73,74]. In this study, the *S. viridis* pollen grain in the Horminum section is oblate-spheroidal. When this study is evaluated regarding palynological results, it was concluded that pollen morphology characteristics of species were generally similar to each other. Pollen morphological characteristics were not distinguishable in taxonomy in the identification of *Salvia* species observed in this study since there was no discernible variation in the palynological characteristics of the taxa analyzed. This view is supported by some other studies [73,74,75,76].

## 5. Conclusions

The chemotaxonomic study showed that the essential oil of *Salvia* species varies slightly depending on ecological, climatic, plant collection periods, and location. However, it is also a fact that the amounts of major common constituents in *Salvia* species vary depending on the species of the species. In other words, the fact that the constituents in *Salvia* species are generally similar, without the effect of harvest time and locality, makes it possible to standardize the essential oils of *Salvia* species. The detailed characterization of oil composition carried out in this study has revealed the existence of various valuable compounds in the selected *Salvia* species, demonstrating their applicability for medicinal and pharmaceutical purposes as well as in the cosmetic and beverage industries. According to the experimental results, it was found to have antimicrobial activity against all tested microorganisms at certain rates. It is believed that the strong antimicrobial effect is due to these valuable chemical components. New research should be carried out on other *Salvia* species and in new areas, and new data should be obtained by conducting practical experiments on the antimicrobial and antibacterial effects of *Salvia* species on rats.

Additionally, the morphological, anatomical, and palynological information gleaned from this research will serve as a foundation for biosystematic analyses of *Salvia* species.

## Figures and Tables

**Table 1:** Locality information of *Salvia* species.

Species	Locality	Collecter
*S. absconditiflora*	B7 Elazığ to Malatya 40 km, 17.06.2019, altitude 1200 m	A. Demirpolat 1123
*S. ceratophylla*	B7 Elazığ to Malatya 20 km steppe, 25.05.2019, altitude of 1400 m	A. Demirpolat 1144
*S. multicaulis*	B8 Bingol to Elazığ 45 km, 10.06.2019, altitude of 1250–1350 m	A. Demirpolat 1167
*S. syriaca*	B7: Elazığ-Baskil fields and wastelands 10.06.2019, 1220–1300 m	A. Demirpolat 1165
*S. verbenaca*	B8: West of Sancak upland slopes, 29.05.2019, altitude of 1250–1300 m	A. Demirpolat 1289
*S. viridis*	B8: Elazig-Bingöl 65. km, fields and wastelands, 17.06.2019 altitude 900–950 m	A. Demirpolat 1128

**Table 2:** Essential oils chemical composition of six species the *Salvia*.

*No*	*Component*	*RI*	*RI* (*lit*) (±)	*RT*	*IM*	*S1*	*S2*	*S3*	*S4*	*S5*	*S6*
1.	*α*-Thujene	916	917 [33]	11.196	RI, MS	3.09	2.45	1.28	0.49	0.09	2.50
2.	*α*-Pinene	938	938 [33]	11.498	RI, MS	**5.62**	3.76	0.55	1.21	0.57	2.25
3.	Camphene	1035	1035 [23]	12.157	RI, MS	0.86	1.39	2.71	2.34	0.23	2.38
4.	*β*-Pinene	973	973 [33]	13.521	RI, MS	2.50	1.98	2.06	1.04	**7.21**	**6.31**
5.	*β*-Myrcene	990	990 [33]	14.342	RI, MS	0.90	-	0.37	0.45	0.08	1.45
6.	*α*-Phellandrene	1004	1004 [33]	14.929	RI, MS	4.97	-	1.57	0.13	0.23	0.43
7.	Limonene	1029	1029 [33]	16.184	RI, MS	0.51	0.56	-	1.23	0.19	1.54
8.	1,8-Cineol	1095	1033 [33]	16.292	RI, MS	**17.94**	**12.98**	**11.99**	**11.45**	**14.06**	3.24
9.	*γ*-Terpinene	1060	1060 [33]	17.715	RI, MS	1.53	-	-	0.56	0.15	0.32
10.	Linalool	1145	1148 [23]	19.849	RI, MS	0.94	0.78	1.09	-	0.36	-
11.	Camphor	1185	1185 [23]	22.017	RI, MS	4.97	3.67	-	-	1.46	1.46
12.	n-Decanal	1185	1204 [33]	22.957	RI, MS	-	-	-	-	0.14	0.14
13.	**Borneol**	1200	1199 [23]	23.165	RI, MS	**10.4**	3.56	**5.74**	**11.0**	**7.02**	9.65
14.	Terpinen-4-ol	1205	1179 [33]	23.738	RI, MS	-	-	-	-	-	0.09
15.	Terpinolene	1210	1193 [33]	24.426	RI, MS	-	0.45	0.34	0.21	-	1.57
16.	Myrtenol	1216	1216 [23]	24.644	RI, MS	-	1.78	2.98	-	2.09	0.06
17.	Thymol	1297	1297 [34]	29.440	RI, MS	1.39	2.75	-	1.92	1.45	4.76
18.	Carvacrol	1300	1317 [34]	29.913	RI, MS	-	-	0.21	-	2.08	1.34
19.	*α*-Cubebene	1323	1337 [23]	32.040	RI, MS	-	-	0.67	0.34	-	-
20.	Eugenol	1345	1359 [33]	32.391	RI, MS	1.23	-	-	0.98	0.21	0.22
21.	*α*-Copaene	1352	1376 [34]	33.276	RI, MS	1.39	2.71	0.87	0.31	0.94	3.92
22.	*δ*-Cadinene	1358	1529 [33]	35.663	RI, MS	1.45	1.76	1.54	-	-	0.30
23.	5,9-Undecadien	1411	1411 [23]	35.922	RI, MS	-	0.35	-	-	-	-
24.	**Caryophyllene**	1424	1424 [33]	36.100	RI, MS	**8.45**	**8.36**	**8.51**	4.95	**15.01**	2.29
25.	**Bicyclogermacrene**	1443	1445 [23]	36.112	RI, MS	1.46	2.65	**5.89**	**11.03**	3.66	**6.93**
26.	*α*-Humulene	1418	1418 [23]	36.762	RI, MS	0.56	1.33	2.41	-	1.25	-
27.	Isobornil asetat	1467	-	37.231	RI, MS	0.78	0.59	-	-	0.98	0.98
28.	1,5-Epoxy*Salvia*l-4[14]-ene	1490	1490 [23]	37.542	RI, MS	1.72	3.91	4.64	3.97	1.22	**6.83**
29.	*γ*-Cadinene	1514	1511 [34]	37.735	RI, MS	0.01	0.42	0.49	0.35	0.28	0.31
30.	Isolongifolene	1518	1517 [23]	38.081	RI, MS	-	-	-	-	-	0.65
31.	*β*-Selinene	1521	1441 [23]	38.176	RI, MS	0.45	0.29	0.75	0.71	0.58	0.45
32.	Germacrene B	1562	1524 [23]	40.349	RI, MS	0.14	1.20	0.24	3.76	-	0.25
33.	*α*-Curcumene	1569	1483 [34]	40.489	RI, MS	0.18	4.89	0.20	-	1.04	-
34.	**Spathulenol**	1572	1571 [34]	42.036	RI, MS	**9.09**	**20.13**	**18.10**	**13.18**	**11.42**	**9.35**
35.	**Caryophyllene oxide**	1595	1578 [34]	42.241	RI, MS	**10.14**	**14.68**	**17.20**	**16.15**	**16.18**	**17.54**
36.	Benzene	1598	-	43.041	RI, MS	0.16	0.58	0.13	5.66	0.38	1.41
37.	Aromadendrene oxide	1650	1650 [35]	44.453	RI, MS	0.26	1.11	0.07	0.84	0.98	-
**Total**						**100**	**98.62**	**92.60**	**94.26**	**91.54**	**93.83**

RI: Retention Indices; RI(lit): The Retention Indices literature, RI: based on retention index; MS: based on mass spectra matching; RT: Retention Time; IM: Identification method, *S1*: *S. absconditiflora*, *S2*: *S. ceratophylla*, *S3*: *S. multicaulis*, *S4*: *S. verbenaca*, *S5*: *S.viridis*, *S6*: *S. syriaca*.

**Table 3:** Pollen morphological data of the studied *Salvia* species.

*Species*	P (μ)	E (μ)	P/E	Ornamentation	Clg (μ)	Clt (μ)	Ex (μ)	Ap (μ)
***S. absconditiflora***	42.1 ± (0.7)	50.0 ± (1.8)	0.84 Suboblate	Reticulate	28.1 ± (1.1)	5.9 ± (0.5)	1.9 ± (0.2)	6.3 ± (2.7)
***S. ceratophylla***	38.2 ± (2.5)	43.5 ± (2.8)	0.87 Suboblate	Reticulate	25.3 ± (2.0)	4.2 ± (2.8)	1.6 ± (0.3)	5.9 ± (2.4)
***S. multicaulis***	57.2 ± (2.7)	55.3 ± (1.2)	1.03 Prolate-spheroidal	Reticulate	38.6 ± (3.1)	6.4 ± (0.9)	1.7 ± (0.3)	5.7 ± (1.0)
***S. verbenaca***	34.2 ± (0.6)	29.2 ± (1.2)	1.17 Subprolate	Reticulate	23.2 ± (0.9)	2.5 ± (1–2)	1.2 ± (0.6)	7.2 ± (1.5)
***S. viridis***	39.1 ± (2.0)	44.4 ± (3.3)	0.88 Oblate-Spheroidal	Reticulate	25.7 ± (2.7)	5.7 ± (1.2)	1.3 ± (0.3)	6.4 ± (2.6)
***S. syriaca***	36.8 ± (3.3)	31.7 ± (3.0)	1.16 Subprolate	Reticulate	24.6 ± 3.9	6.3 ± (1.8)	1.6 ± (0.3)	5.0 ± (2.3)

**Table 4:** Morphological and morphometrical characters of *Salvia* species.

	*S.* *absconditiflora*	*S.* *ceratophylla*	*S.* *multicaulis*	*S.* *verbenaca*	*S.* *viridis*	*S.* *syriaca*
**Plant stem (cm)**	19–40	30–60	10–45	10–55	7–45	25–55
**Hairs of stem**	Glandular-Dendroid hairs and sessile glands	Glandular-villous densely above	Glandular-pilose to villous	Eglandular-pilose on below, glandular pilose on stems above	Glandular or eglandular pilose	Eglandular- pubescent below, denser above
**Leaf shapes**	Pinnatifid oblong	Pinnatifid oblong	Pinnatifid Ovate to suborbicular	Pinnatifid Oblong to ovate	Pinnatisect Oblong ovate	Linear, oblong to ovate
**Width of the leaf (cm)**	1–3	4–8	1–4	1.5–7	1.5–2.5	2.5–5
**Length of leaf (cm)**	1–6	12–25	2–6	2–10	1–3	2–9.5
**Petiole (cm)**	0.5–3	6–18	1.5–6	1.2–8	2–5	3–6
**Bracts (mm)**	12 × 10 ovate	12 × 16 ovate	15 × 10 ovate	5 × 5 ovate-acuminate	6 × 10 ovate	5 × 5 ovate
**Inflorescences**	Verticillaster	Paniculate	Verticillaster	Verticillaster	Verticillaster	Verticillaster
**Flowered**	3–5	2–5	4–10	4–6	2–5	4–6
**Colors of calyx**	Yellowish- Green	Yellowish-Green	Green	Yellowish- Green	Green	Yellowish- Green
**Corolla size (mm)**	15–20	15–20	15–18	12–16	12–15	8–10
**Colors of corolla**	Light Pink	Lilac	Lilac	Dark Purple	Purple to white	White

**Table 5:** Comparative anatomical measurements of investigated *Salvia* species **(μ)**.

	*S.* *absconditiflora*	*S.* *ceratophylla*	*S.* *multicaulis*	*S.* *verbenaca*	*S.* *viridis*	*S.* *syriaca*
***Stem***						
**Cortex layers**	3–4	2–4	2–4	2–4	3–7	3–5
**Collenchyma layers**	6–8	4–8	6–8	2–10	4–7	4–6
**Phloem layers**	3–5	3–5	3–5	2–5	2–5	4–9
**Xylem layers**	5–10	3–10	3–10	3–11	6–14	8–17
***Root***						
**Periderm layers**	2–5	3–4	2–4	2–4	3–5	2–5
**Periderma thickness (μm)**	9–18	6–20	10–28	6–13	7–16	9–21
**Cortex layers**	6–15	10–14	12–18	10–17	12–20	4–9
**Sclerenchyma layer**	3–5	3–5	3–5	4–8	2–3	3–6
**Pith region**	10–17	9–15	10–18	8–15	1–3	11–17
***Petiole***						
**Petiole shape**	Triangular	D-shaped	Triangular	U-shaped with obtuse margins	U-shaped	D-shaped
**Collenchyma cell layers**	2–4	3–7	4–7	3–7	3–6	3–7
**Sclerenchyma layer**	4–8	5–12	1–3	3–5	2–3	4–10

## Data Availability

Not applicable.

## References

[1] Minhui L., Qianquan L., Chunhong Z., Na Z., Zhanhu C., Luqi H., Peigen X. (2013). An ethnopharmacological investigation of medicinal *Salvia* plants (Lamiaceae) in China. Acta Pharm. Sin..

[2] Celep F. (2010). Revision of the Genus *Salvia* L. (Labiatae) in the Mediterranean and The Aegean Geographic Regions of Turkey. Ph.D. Thesis.

[3] Karakuş M., Baydar H., Erbaş S. (2017). Tıbbi Adaçayı (*Salvia officinalis* L.) Populasyonundan Geliştirilen Klonların Verim ve Uçucu Yağ Özellikleri. J. Field Crops Cent. Res. Inst..

[4] Bahadırlı N.P. (2014). Hatay İlinde Doğal Olarak Yetişen Adaçayı (*Salvia* Spp.) Populasyonlarının Ssr Markörleri İle Moleküler Karakterizasyonu ve Sitogenetik Analizleri. Master’s Thesis.

[5] Celep F., Raders E., Drew B. (2020). Two new hybrid species of Salvia (S.× karamanensis and S.× doganii) from Turkey: Evidence from molecular and morphological studies. Turk. J. Bot..

[6] Davis P.H. (1982). Flora of Turkey and The East Aegeans Islands.

[7] Vural M., Adıgüzel N. (1996). A new species from Central Anatolia: *Salvia aytachii* M. Vural et N. Adıgüzel (Labiatae). Turk. J. Bot..

[8] Dönmez A.A. (2001). A new species of *Salvia* (Lamiaceae). Bot. J. Linn. Soc..

[9] Walker J.B., Sytsma K.L. (2007). Staminal evolution in the genus *Salvia* (Lamiaceae): Molecular phylogenetic evidence for multiple origins of the staminal lever. Ann. Bot..

[10] Sancar P.Y., Demirpolat A., Cacan E. (2021). Determination of Genetic Sthisces of Some Alfalfa Taxa (*Medicago sativa* L.) Cultured in Turkey. Fresenius Environ. Bull..

[11] Sancar P.Y., Tukur U., Civelek S., Kursat M. (2021). The molecular investigations on the subgenus *Artemisia* Less. of the genus *Artemisia* L. (Asteraceae) in Turkey. Braz. J. Biol. Inst. Int. De Ecol..

[12] Hayta S., Dogan G., Yüce E., Bagci E. (2015). Composition of the essential oil of two *Salvia* taxa *Salvia sclarea* and *Salvia verticillata* subsp. verticillata from Turkey. Nat. Sci. Discov..

[13] Sancar P.Y. (2021). Çeşitli Bitki Taksonlarında Bazı DNA İzolasyon Yöntemlerinin Karşılaştırmalı Analizi. Uluslararası Doğu Anadolu Fen Mühendislik Ve Tasarım Derg..

[14] Shanaida M., Hudz N., Białoń M., Kryvtsowa M., Svydenko L., Filipska A., Wieczorek P.P. (2021). Chromatographic profiles and antimicrobial activity of the essential oils obtained from some species and cultivars of the Mentheae tribe (Lamiaceae). Saudi J. Biol. Sci..

[15] Soltanbeigi A., Yıldız M., Dıraman H., Terzi H., Sakartepe E., Yıldız E. (2021). Growth responses and essential oil profile of *Salvia officinalis* L. Influenced by water deficit and various nutrient sthisces in the greenhouse. Saudi J. Biol. Sci..

[16] Kamatou G.P.P., Makunga N.P., Ramogola W.P.N., Viljoen A.M. (2008). South African *Salvia* species: A review of biological activities and phytochemistry. J. Ethnopharmacol..

[17] Kilic O. (2016). Chemical Composition of Fthis *Salvia* Species from Turkey, a Chemotaxonomic Approach. J. Essent. Oil Bear. Plants.

[18] Ulubelen A. (2003). Cardioactive and antibacterial terpenoids from some *Salvia* species. Phytochemistry.

[19] Ahmad M., Qureshi R., Arshad M., Khan M.A., Zafar M. (2009). Traditional herbal remedies used for the treatment of diabetes from district Attock (Pakistan). Pak. J. Bot..

[20] Şenol F.S., Orhan İ., Celep F., Kahraman A., Doğan M., Yılmaz G., Şener B. (2010). Survey of 55 Turkish *Salvia* taxa for their acetylcholinesterase inhibitory and antioxidant activities. Food Chem..

[21] Ahmad M., Khan M.P.Z., Mukhtar A., Zafar M., Sultana S., Jahan S. (2016). Ethnopharmacological survey on medicinal plants used in herbal drinks among the traditional communities of Pakistan. J. Ethnopharmacol..

[22] Moretti M.D.L., Peana A.T., Satta M.A. (1997). A study of antiinflammatory and peripheral analgesic actions of *Salvia sclarea* oil and its main constituents. J. Essent. Oil. Res..

[23] Doğan G., Hayta Ş., Demirpolat A., Bağcı E. (2017). Composition of The Essential Oil of Endemic *Salvia cryptantha* (Lamiaceae) Montbret & Aucher Ex Bentham From. Hacettepe J. Biol. Chem..

[24] Hisarlı N.D. (2013). Effect of *Salvia absconditiflora* Extract on the Gene Expressions of Gsto and Gstz in Mcf-7 And Mda-Mb-231 Cells. Ph.D. Thesis.

[25] Flamini G., Cioni P.L., Morelli I., Bader A. (2005). Essential oils of the aerial parts of three *Salvia* species from Jordan: *Salvia lanigera*, spinosa and *S. syriaca*. Food Chem..

[26] Darwish M.A., Cabral C., Ali Z., Wang M., Khan S., Jacob M., Jain S.K., Tekwani B., Zulfigar F., Khan I. (2020). *Salvia ceratophylla* L. from South of Jordan: New insights on chemical composition and biological activities. Nat. Prod. Bioprospect..

[27] Askun T., Tumen G., Satil F., Ates M. (2009). Characterization of the phenolic composition and antimicrobial activities of Turkish medicinal plants. J. Pharm. Biol..

[28] Askun T., Baser K.H.C., Tümen G., Kürkcüoglu M. (2010). Characterization of essential oils of some *Salvia* species and their antimycobacterial activities. Turk. J. Biol..

[29] Kahraman A., Doğan M. (2010). Comparative study of *Salvia limbata* C.A. and *S. palaestina* Bentham (sect. Aethiopis Bentham, Labiatae) from East Anatolia, Turkey. Acta Bot. Croat..

[30] Erdtman G. (1945). Pollen morphology and plant taxonomy 4. Labiatae, Verbenaceae and Avicenniaceae. Sven. Bot. Tidskr..

[31] Cantino P.D., Harley R.M., Wagstaff S.J., Harley R.M., Reynolds T. (1992). Genera of Labiatae: Status Classification. Advanced in Labiatae Science.

[32] Silva E.A.J.D., Silva V.P.D., Alves C.C.F., Alves J.M., Souchie E.L., Barbosa L.C.D.A. (2016). Harvest time on the content and chemical composition of essential oil from leaves of guava. Ciência Rural.

[33] Ayoub I.M., Abdel-Aziz M.M., Elhady S.S., Bagalagel A.A., Malatani R.T., Elkady W.M. (2022). Valorization of *Pimenta racemosa* Essential Oils and Extracts: GC-MS and LC-MS Phytochemical Profiling and Evaluation of Helicobacter pylori Inhibitory Activity. Molecules.

[34] Hazzit M., Baaliouamer A., Faleiro M.L., Miguel M.G. (2006). Composition of the essential oils of *Thymus* and *Origanum* species from Algeria and their antioxidant and antimicrobial activities. J. Agric Food. Chem..

[35] Reza G.H., Ebrahim S., Hossein H. (2007). Analysis by gas chromatography—Mass spectrometry of essential oil from seeds and aerial parts of *Ferulago angulata* (Schlecht.) Boiss gatheres in Nevakoh and Shahoo, Zagross mountain, west of Iran. Pak. J. Biol. Sci..

[36] Horváth G., Bencsik T., Ács K., Kon K., Rai M. (2016). Chapter 12—Sensitivity of ESBL-Producing Gram-Negative Bacteria to Essential Oils, Plant Extracts, and Their Isolated Compounds. Antibiotic Resistance.

[37] Tekin M. (2022). A morphological, anatomical and palynological study on *Aethionema lepidioides* (Brassicaceae) an endangered and endemic species to Turkey. Acta Bot. Croat..

[38] Davis A.P., Barnett J.R. (1997). The leaf anatomy of the genus *Galanthus* L. (Amaryllidaceae J. St.-Hil.). Bot. J. Linn. Soc..

[39] Faegri K., Iversen J. (1975). Textbook of Pollen Analysis.

[40] Ertman G. (1952). Pollen Morphology and Plant Taxonomy Angiosperms.

[41] Kılıç N., Yılmaz Dağdeviren R., Caner H., Akkemik Ü. (2020). Türkiye’de Kullanılmakta Olan Palinoloji ve Polen Terimleri Üzerine Bir Değerlendirme ve Öneriler. Avrasya Terim Derg..

[42] Majeed S., Zafar M., Ahmad M., Kilic O., Sultana S., Raza J., Jabeen M. (2020). Pollen morphological investigations of family Cactaceae and its taxonomic implication by light microscopy and scanning electron microscopy. Microsc. Res. Tech..

[43] Ertas A., Akdeniz M., Yener I., Ozturk M., Tokul Olmez O., Firat M., Kolak U. (2022). Essential oil, aroma, and fatty acid profiles of five endemic *Salvia* taxa from Turkey with chemometric analysis. Chem. Biodivers..

[44] Baran P. (2005). *Salvia argentea* L. ve *Salvia viridis* L. (Lamiaceae) Türleri Üzerinde Morfolojik ve Anatomik bir Araştırma. Ph.D. Thesis.

[45] Gursoy N., Tepe B., Akpulat H.A. (2012). Chemical composition and antioxidant activity of the essential oils of *Salvia palaestina* (Bentham) and *S. ceratophylla* (L.). Rec. Nat. Prod..

[46] Bagci E., Koçak A. (2008). *S. palaestina* ve *S. tomentosa* Türlerinin Uçucu Yag Kompozisyonu, Kemotaksonomik Bir Yaklasim Fırat Üniv. J. Firat Univ..

[47] Mirza M., Sefidkon F. (1999). Essential oil composition of two *Salvia* species from Iran, *Salvia nemorosa* L. and *Salvia reuterana* Boiss. Flav. Frag. J..

[48] Fahimeh S., Mazooji A., Darzikolaei S.A. (2011). Chemotaxonomy of six *Salvia* species using essential oil composition markers. J. Med. Plants Res..

[49] Sonboli A., Babakhani B., Mehrabian A.R. (2006). Antimicrobial Activity of Six Constituents of Essential Oil from *Salvia*. Z. Naturforsch..

[50] Chialva F., Monguzzi F., Manitto P. (1999). Composition of Five *Salvia* species. J. Essent. Oil Res..

[51] Demirpolat A. (2022). *Salvia syriaca* L. Türünün Uçucu Yağ Kompozisyonu. Uluslararası Gıda Tarım ve Hayvan Bilimleri Dergisi.

[52] Özdemir E. (2015). In Vitro Genotoxicity of 1,8-Cineole (Eucalyptol) Compound Effects. Master’s Thesis.

[53] Şimşek M., Duman R. (2017). Investigation of Effect of 1,8-cineole on Antimicrobial Activity of Chlorhexidine Gluconate. Pharmacogn. Res..

[54] Wichtel M. (2002). Teedrogen und Phytopharmaka.

[55] DeBarber A.E., Bleyle L., Roullet J.B., Koop D.R. (2004). w-Hydroxylation of farnesol by mammalian cytochromes P450. Biochim. Biophys. Acta.

[56] Tan N., Satana D., Sem B., Tan E., Altan H.B., Demirci B. (2016). Antimycobacterial and antifungal activities of selected four *Salvia* species. Rec. Nat. Prod..

[57] Ziaei A., Ramezani M., Wright L., Paetz C., Schneider B., Amirghofran Z. (2011). Identification of spathulenol in *Salvia mirzayanii* and the immunomodulatory effects. Phytother. Res..

[58] Cantrell C.L., Klun J.A., Bryson C.T., Kobaisy M., Duke S.O. (2005). Isolation and identification of mosquito bite deterrent terpenoids from leaves of American (*Callicarpa americana*) and Japanese (*Callicarpa japonica*) beautyberry. J. Agric. Food Chem..

[59] Knobloch K., Pauli A., Iberl B., Wegand H., Weis N. (1989). Antibacterial and Antifungal Properties of Essential Oil Components. J. Essent. Oil Res..

[60] Buchbauer G., Jager W., Jirovetz L., Meyer F., Dietrich F. (1992). Effects of Valerian Root Oil, Borneol, Isoborneol, Bornyl acetate and Isobornyl acetate on the Motility of Laboratory Animals (mice) After Inhalation. Pharmazie.

[61] Zhang Q.L., Bingmei M.F., Zhang J.Z. (2017). Borneol, a novel agent that improves central nervous system drug delivery by enhancing blood–brain barrier permeability. Drug Deliv..

[62] Zou L., Lin L., Hu H.L., Wang Y., Wang P., Zhao G., Wang Z.-G. (2012). Effect of borneol on intestinal absorption of muscone in rats. China J. Chin. Mater. Med..

[63] Yiğit D., Kandemir A., Yiğit N. (2002). Antimicrobial Activity of Some Endemic Plants (*Salvia cryptantha, Origanum acutidens, Thymus sipyleus* ssp. *sipyleus*). Erzincan Üniversitesi Eğitim Fakültesi Derg..

[64] Yilmaz İ., Bülbül A.S., Kocabaş Y.Z. (2022). *Salvia ceratophylla* L. and *Ricotia aucheri* (Boiss.) B.L. İnvestigation of Biological and Cytotoxic Activities of Burtt Plants in Vitro Conditions. Biyol. Bilim. Araştırma Derg..

[65] Paknejadi M., Foroohi F., Yousefzadi M. (2012). Antimicrobial activities of the essential oils of five *Salvia* species from. J. Paramed. Sci..

[66] Yousefzadi M., Sonboli A., Karimi F., Ebrahimi S.N., Asghari B., Zeinali A. (2007). Antimicrobial activity of some *Salvia* species essential oils from Iran. Z. Für Nat..

[67] Pehlivan M., Sevindik M. (2018). Antioxidant and Antimicrobial Activities of *Salvia multicaulis*. Türk Tarım Gıda Bilim Ve Teknol. Derg..

[68] Sarac N., Uğur A. (2007). Antimicrobial activities and usage in folkloric medicine of some Lamiaceae species growing in Mugla, Turkey. Eur. Asia J. Bio Sci..

[69] Özdemir C., Şenel G. (1999). The Morphological, Anatomical and Karyological Properties of *Salvia sclarae* L. Turk. J. Bot..

[70] Çobanoğlu D. (1987). *Salvia palestina* Bentham’ın Morfolojik Ve Sitolojik Özellikleri. Doğa Tr. Bot..

[71] Metcalfe C.R., Chalk L. (1972). Anatomy of Dicotyedon.

[72] Kahraman A. (2011). Morphology, Anatomy and Systematics of the Genus *Salvia* L. (Lamiaceae) in East Southeast Anatolia, Turkey. Ph.D. Thesis.

[73] Özler H., Pehlivan S., Kahraman A., Doğan M., Celep F., Başer B., Yavru A., Bagherpthis S. (2011). Pollen morphology of the genus *Salvia* L. (Lamiaceae) in Turkey. Flora.

[74] Özler H., Pehlivan S., Celep F., Dogan M., Kahraman A., Yavru-Fişne A., Baser B., Bagherpthis S. (2013). Pollen morphology of Hymenosphace and Aethiopssection of the genus *Salvia* L. (Lamiaceae) in Turkey. Turk J. Bot..

[75] Kılıç F.M. (2021). Pollen Morphological Investigations of *Salvia* L. In Southeastern of Turkey and Its Taxonomic Implication. Bangladesh J. Plant Taxon..

[76] Moon H.K., Vinckier S., Walker J.B., Smets E., Huysmans S.A. (2008). Search for phlogenetically informative pollen characters in the subtribe Salviinae (Mentheae: Lamiaceae). Int. J. Plant Sci..

